# Geometry-Based Distributed Spatial Skyline Queries in Wireless Sensor Networks

**DOI:** 10.3390/s16040454

**Published:** 2016-03-29

**Authors:** Yan Wang, Baoyan Song, Junlu Wang, Li Zhang, Ling Wang

**Affiliations:** 1 School of Information, Liaoning University, Shenyang 110036, China; wang_yan@lnu.edu.cn (Y.W.); wangjunlu@lnu.edu.cn (J.W.); 15846142643li@gmail.com (L.Z.); 15702481669ling@gmail.com (L.W.); 2 School of Information Science and Engineering, Northeastern University, Shenyang 110819, China

**Keywords:** wireless sensor network, environmental monitoring, distributed spatial skyline query, convex hull, cutting node

## Abstract

Algorithms for skyline querying based on wireless sensor networks (WSNs) have been widely used in the field of environmental monitoring. Because of the multi-dimensional nature of the problem of monitoring spatial position, traditional skyline query strategies cause enormous computational costs and energy consumption. To ensure the efficient use of sensor energy, a geometry-based distributed spatial query strategy (GDSSky) is proposed in this paper. Firstly, the paper presents a geometry-based region partition strategy. It uses the skyline area reduction method based on the convex hull vertices, to quickly query the spatial skyline data related to a specific query area, and proposes a regional partition strategy based on the triangulation method, to implement distributed queries in each sub-region and reduce the comparison times between nodes. Secondly, a sub-region clustering strategy is designed to group the data inside into clusters for parallel queries that can save time. Finally, the paper presents a distributed query strategy based on the data node tree to traverse all adjacent sensors’ monitoring locations. It conducts spatial skyline queries for spatial skyline data that have been obtained and not found respectively, so as to realize the parallel queries. A large number of simulation results shows that GDSSky can quickly return the places which are nearer to query locations and have larger pollution capacity, and significantly reduce the WSN energy consumption.

## 1. Introduction

Nowadays applications using the sensor network monitoring strategy are being more and more widely used, such as in forest fire monitoring systems, CitySee, real time CO_2_ monitoring systems, real time shortest path for drivers [1], a complex embedded system-CPS [2], graph similarity issue [3], digital library services [4], data security [5], the development of the Internet of Things [6,7], the development of the Web of Things [8], Semantic Link Network (SLN) [9,10], different bird species’ behavior research, air quality inspection [11] and so on. Big data analytics for these applications has become more and more essential [12,13]. In terms of the air quality inspection situation, in European and American countries, monitoring stations are mostly set near big cities, and reflect the average air quality level of a whole region. In China, the current information people get is from placing in cities several monitoring sub-stations, which locations must be set in places which are widely apart and not disturbed by people, rather than in places near to the pollution sources. This deployment can also reflect the average air quality level of a city. The above traditional methods can’t monitor the pollution around places with dense populations, and they are always set up to monitor a wide range and will be limited in flexibility when an arbitrarily designated area is queried. Therefore, it is very necessary to apply the spatial skyline query method in wireless sensor networks used for air quality monitoring [14,15,16].

As traditional monitoring strategies can only monitor the average situation of a large area and have limited flexibility for any small range, this paper proposes a skyline query method for sensor networks. Considering that environmental monitoring involves more spatial attributes, and often incurs a lot of computational cost for general skyline queries, we propose the geometry-based distributed spatial skyline query method in wireless sensor networks (GDSSky). The strategy can quickly find the locations which are near to the query places and have higher pollution potential, and it also can reduce the energy cost.

The paper is an extension of our previous work Geometry-Based Spatial Skyline Query in Wireless Sensor Networks [17]. It adds a more detailed description, new experiments and a new distributed parallel strategy and query strategy. The major contributions of this paper are as follows:

- We design the cut of skyline region based on convex hull vertices method, which can cut out a lot of the non-skyline region by the rectangular B strategy and reduce the comparison times between nodes and improve the efficiency.
- We propose a distributed query method based on the data node tree concept to traverse all the neighbor sensor monitoring regions, and enter them into the queue according to the distance by the monotone function, so it can implement the execution in parallel.
- We design a clustering strategy for the parallel execution of general skyline queries on the non-spatial attributes of the spatial skyline, and conduct the spatial skyline query on the remaining spatial skyline points which are still not found by the tree method at the same time, so we can implement a distributed execution between different sub-regions.
- We propose the concepts of cuts among sub-regions and cuts in sub-regions for the collection. This can reduce the data transmissions in the network and reduce the sensor energy cost.

The rest of the paper is organized as follows: Section 2 discusses the related work. Section 3 describes geometry-based spatial skyline queries. Section 4 describes the geometry-based distributed spatial skyline query algorithm. Section 5 discusses the experiments and the performance analysis. Finally, Section 6 concludes this paper with the future work.

## 2. Related Work

### 2.1. The Non-Spatial Skyline Query Method

For the skyline query in the sensor network, the most common method is the cut method based on a filter [18,19,20,21,22,23]. Y Kwon *et al.* [18] set down the initial filter from the root node and updates each node constantly; the strongest cut filter is produced in the leaf node and returned with the query results. In [19], a multidimensional filter is acquired by calculation to improve the cut efficiency of the overall wireless communication between sensor nodes. In another approach [20] a local or global filter is set up at the sensor nodes to restrain unnecessary data transmissions, however, the global filter has some limitations in a large-scale sensor network, as these methods will result in large amounts of transmission consumption in the data recovery process, and cost a lot of storage and transmission costs when calculating the filter. In [21], the authors use a global and local optimization strategy to implement skyline queries for more dimensions, and it associates the sub-space skyline with the parent spatial skyline, but it must merge the sub-space skyline queries into the existing expanded parent spatial skyline query. Undoubtedly this has a computational cost. G Wang *et al.* [22] continuous fragmented skylines over distributed streams which constitute a distributed skyline query method based on streams are presented. A complex skyline monitoring function on the distributed fragmented objects, and a model of the fragments of the object are proposed. B Chen *et al.* [23] face the problem of curse of dimensionality and proposes to acquire truly controlled and characteristic results to select the most interesting objects from among all the skyline objects.

Therefore, in these kinds of strategies, the dominant compare counts and computational cost will increase. The tuple is too long, so the transmission and energy consumption between data will increase. The data nodes may be obtained from severely polluted areas but far from locations crowded with people. However, these nodes are not the geographic locations people really care about.

### 2.2. Spatial Skyline Query Method

Unlike no-spatial skyline queries, the spatial skyline query strategy includes two concepts: the spatial dominance relation and the spatial skyline.

- The spatial dominance relation

**Figure 1:**
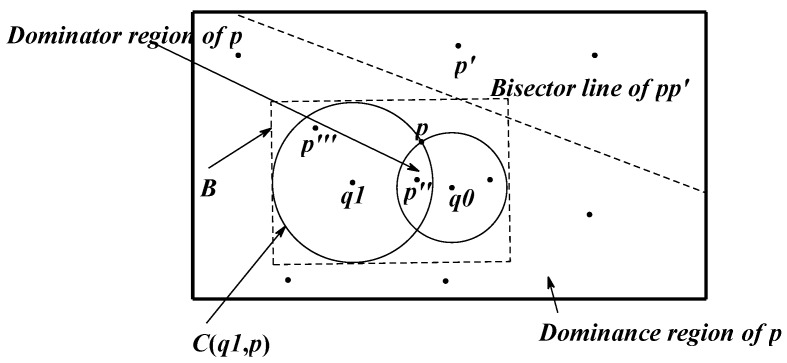
The dominance relation between nodes.

A data node set P = {p1, p2,..., pn} representing the sensor nodes includes some points in 2-dimensional space. Among them, the function of the sensor node is sensing data, transforming data and participating in the skyline query. The query node set Q = {q1,...,qn} shown as the residential areas in Figure 1 includes some points in 2-dimensional space. D(pi, qj) is the distance in 2-dimensional space. The node dominance relation is shown in Figure 1. Among them, rectangle B is the minimum rectangle boundary MBR. In the query process, B updates constantly, B_ {new} = B∩MBR (SR (p, Q)). The interior of B contains all the candidate nodes. Nodes outside B are the dominated ones, so there is no judgment of dominance relation to those.

As shown in the figure, given a 2-dimensional query node set Q = {q1, ..., qn} and two sensor nodes p, p’, the space dominance relation is defined as follows:

*Definition 1*: A point p spatially dominates a point p’ with respect to Q if and only if D (p, qi) ≤ D (p’, qi) for every qi ∈ Q, and D (p, qj) < D(p’, qj) for some qj ∈ Q.

- The spatial skyline

By the comparison between the above dominance relations, we can find the data nodes that are part of the spatial skyline. Given the data node set P = {p1, p2, ..., pn} and the query node set Q = {q1,..., qn}, the definition is as follows:

*Definition 2*: A point p ∈ P is a spatial skyline point with respect to Q if and only if p is not spatially dominated by any other point of P. The node p is a spatial skyline with respect to Q, denoted as p ∈ S(Q). This way we get the formal description of Equation (1) shown as follows:

p ∈ S(Q) ⇔ ∀p’ ∈ P, p’ ≠ p and p’ ∉ S(Q), ∃qi ∈ Q, so that D(p, qi) ≤ D(p’, qi)(1)

In [24,25,26,27,28], the authors describe the existing spatial skyline query methods. These methods are always applied in skyline queries with respect to the query nodes. In [24,25], the authors propose an algorithm for transmitting all the other data node information to one node in which the dominance relations with the existing skyline nodes is judged. This method will affect the correctness of the overall result once the local networks experience problems, and bring about high transmission costs and low efficiency. S Yoon *et al.* [26] propose dividing the query space into several triangle regions by using a triangulation method and conducting the skyline query in each sub-region. However, the arbitrary triangle area must to wait for the local skyline result from the clockwise neighbor triangle area and cannot query in parallel, so it has low efficiency. In [27], there is a range-based skyline query which uses the I-SKY and N-SKY index methods to consider the spatial and non-spatial attributes of the objects at the same time. W Son *et al.* [28] solve the problem of computing the top-k Manhattan spatial skyline query by using the monotonic function. It computes the top-k skyline points in near linear time. The above spatial skyline query methods can only find the regions which have more influence to the query region on the spatial position, but can't find the environmental pollution overview of the query region.

## 3. Geometry-Based Spatial Skyline Queries

In order to reduce the time for comparing between data, a region partition strategy based on geometry according to the sensor nodes deployed at different locations is proposed in this paper. The attributes detected by the sensors include the spatial attributes (*i.e.*, the distance to each query node) and non-spatial attributes (such as PM_2.5_ or SO_2_ concentration in the air), thus, the paper presents a geometry-based spatial skyline query method to quickly query a part of the geographical positions with greater domination ability in spatial distance, then calculate the maximum query boundary formed by query nodes. It proposes a method of cutting the skyline area based on the convex hull vertices, which can quickly find these data node sets near to crowded places in the spatial attribute.

### 3.1. Regional Division Based on Sensor Deployment

**Figure 2:**
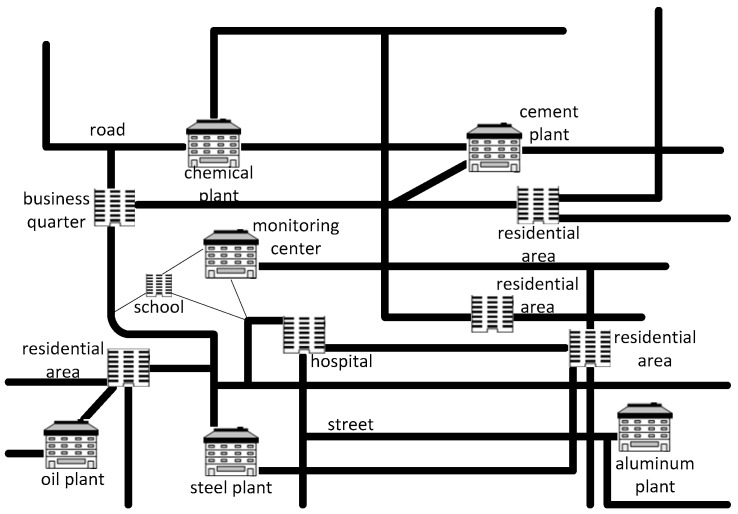
The overview diagram of the city residential area.

As shown in Figure 2, users do not prefer to select one attribute and judge synthetically according to the multiple attribute values. To query the sensor nodes which are closer to the residential areas and where the air pollution level is higher are the skylines we are looking for, so this paper firstly proposes a method for cutting of skyline region based on convex hull vertices to find the data node set closer to the query nodes. The method can quickly return the spatial locations of the above problem which are near to all the crowded regions in the special query range, that is, the nodes having more crowd influence. Then, we further propose a distributed query method based on the data node tree to directly query the non-spatial attributes, such as the values of CO_2_, SO_2_, PM_2.5_ to get the places which are seriously polluted. Above all, we can obtain a source pollution overview which is near to the crowed places by the method of geometry-based distributed spatial skyline queries in wireless sensor networks we propose.

#### 3.1.1. Voronoi Diagram

**Figure 3:**
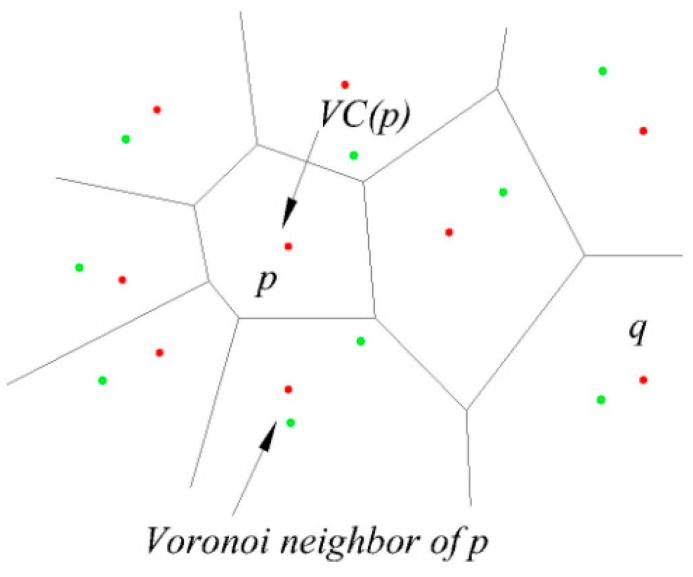
Voronoi diagram.

This paper uses the Voronoi diagram method to divide the spatial region. This method is an efficient geometric partitioning method. Among each sub-region we can judge the dominance relationships between them. As shown in Figure 3, the Voronoi diagram of set P in two-dimensional space divides the space into several regions. For all the points x (where x is a 2-dimensional point) contained in the corresponding region to p (p ∈ P) V (p), we can get the formal description given in Equation (2) as follows:

∀ p’ ∈ P and p’ ≠ P⇒D(x, p) ≤ D(x, p’)(2)

#### 3.1.2. Delaunay Graph

**Figure 4:**
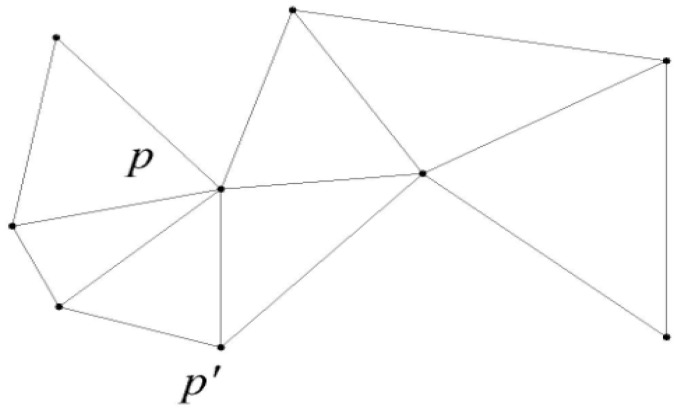
Delaunay graph.

The Delaunay graph is the Voronoi diagram of a dual graph, used to traverse the adjacent Voronoi cell. Figure 4 shows the Delaunay graph corresponding to Figure 3. In a graph G (V, E), V refers to the vertices, E refers to the set of edges. We set V = P, for any two points p, p’ of V, if and only if p is the Voronoi neighbor of p’ in the Voronoi diagram of P, there will be a connection edge of the two points in G. We say graph G is P’s Delaunay graph [29]. Any Delaunay graph of a point set is a planar graph, and it is connected.

#### 3.1.3. The Region Division Method

**Figure 5:**
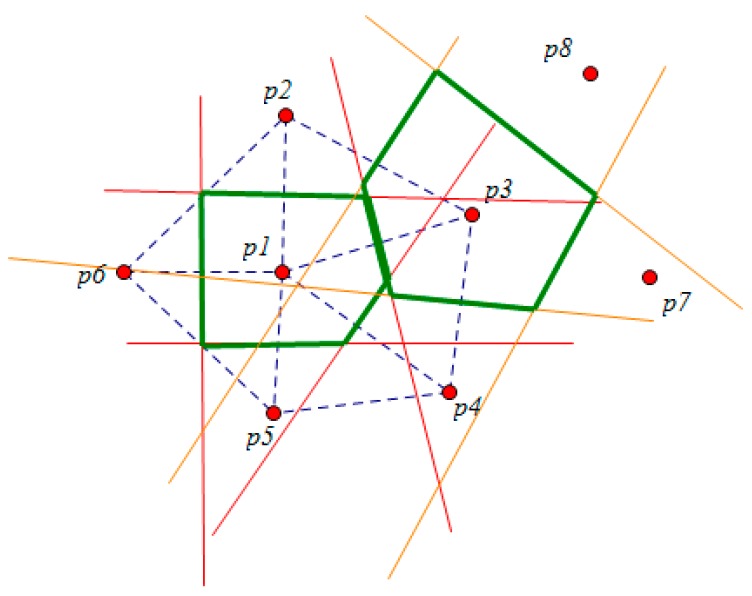
Regional division based on the Voronoi diagram.

Figure 5 is the local map, which contains eight data nodes in the local query region. For example, as a node p1 and its Voronoi cell V (p1), we respectively connect the point p1 with its adjacent nodes in the graph, which are p2, p3 and p4, p5 and p6. For the above five lines, we draw the corresponding perpendicular bisectors, to constitute a polygon made up of these perpendicular bisectors (that is, the bold line polygon in the figure). This polygon is the Voronoi cell V (p1) of the node p1, and any location is represented by the p1, that is to say P1 can represent all of geographic information in the V (p1). In this way data nodes that users want to query and are close to all query nodes, can be judged by the dominance relationship of these data nodes like p1 to gain. This is also an important factor behind why we use the spatial skyline query method based on the geometry in this paper. With this method we can quickly and accurately cut dominated data nodes and query those required data nodes that are the query points close to all query nodes. As shown in the figure, V (p3) of the point p3 has the same formation process as V (p1) of p3, similarly, all other data nodes in this figure can construct their own Voronoi cells this way, so this will form all data nodes’ Voronoi diagram in the whole query region.

**Figure 6:**
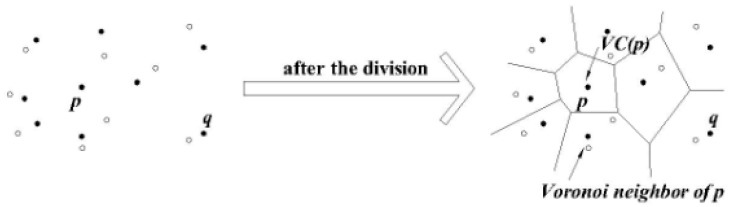
The abstract figure after query division.

Figure 6 is the abstract graph of Figure 5 after regional division based on the Voronoi diagram. The solid dots represent the sensors in the figure, we call the data nodes, and hollow points represent residential areas, called the query nodes. Through the regional division strategy based on the Voronoi diagram, we can quickly judge the positional relationship between a Voronoi cell and the query point, and thus to obtain the spatial skyline fast.

### 3.2. Cutting of Skyline Regions Based on Convex Hull Vertices

In order to improve the spatial skyline query process and accelerate the cutting process of the non-spatial skyline, we propose a reduction method which based on the convex hull vertices of the contour region in this paper. It uses the geometry knowledge, by judging whether the data nodes and the convex hull vertices form the largest location relationship between the convex polygons, to complete partial data queries of the spatial skyline, and thus query the partial spatial skyline data which meets the demand of users.

#### 3.2.1. Convex Hull

**Figure 7:**
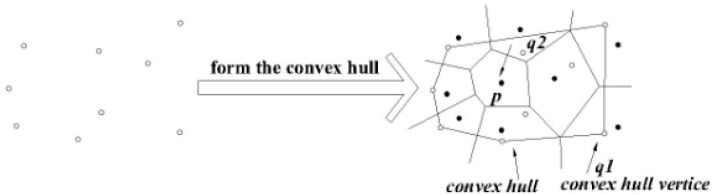
Convex hull.

The query node set on the left side of Figure 6 is denoted as Q and we extract all the hollow points, as shown in Figure 7. The convex hull (denoted as CH) of set Q in 2-dimensional space is the sole smallest convex polyhedron (it is a convex polygon when in the 2-dimensional space), the convex hull contains all points in Q. Each point of the non-convex points does not affect the shape of CH(Q). The symbols used are as follows: the query node set is Q, the query node is denoted as q, the data node set is denoted as P, the data node is denoted as p. If a data point is the closest node from a convex hull query vertex, then it is denoted as p = NN(q) [22], and if a data point is inside the convex hull, then it is denoted as p ∈ CH(Q), the convex hull query vertex in Q is denoted as CHv(Q), the perpendicular bisector line of the line segment pp’ is donated as l ⊥ (pp’). So if p ∈ P and p ∈ S(Q), then S(Q) does not depend on q ∈ Q and q ∉ CHv(Q), we can find that it can greatly improve the efficiency of the algorithm when it is not necessary to calculate the distance to the non-vertex query points in the skyline query.

#### 3.2.2. Cut Method for Skyline Region

In order to query the partial skyline data quickly, we propose a cutting strategy based on the contour region of convex hull vertices in this paper. It can cut out data nodes which are outside the rectangular frame. For determining whether the data node is within the CH (Q), this paper uses a point location query algorithm, which can easily locate a data point whether is inside the query convex hull or not. To reduce the comparison time between data, it proposes an ordering strategy of the monotone function in this paper, to reduce the energy consumption of sensor nodes.

- The point location query

In this phase of the skyline query, in order to determine whether data node and its Voronoi cell are located in the interior of the convex hull, we need a point location query. The point location query is known as a region divided into multiple sub-regions and a query point q appointed by the coordinates, where we need to find the subdomain in which the point q is located. The required output of a point location query usually stays in the sub-region partition containing a given query and the the serial number of the unit. Such as in d dimensional space, what is called the point positioning for a convex polyhedron, there are only two possible search results: one is the query point located inside a polyhedron, the other is located outside the polyhedron. Therefore, the algorithm can be used to easily locate a data point whether it is within the query convex hull or not.

- Cutting method for the skyline region

Each data node’s Voronoi neighbors are known, in other words, the Delaunay graph adjacency list of the point of set P is stored in the neighbor nodes table. Using the point location queries mechanism it will be located in or has crossed the corresponding data at the convex hull of the Voronoi unit node that is the outline of the node.

**Figure 8:**
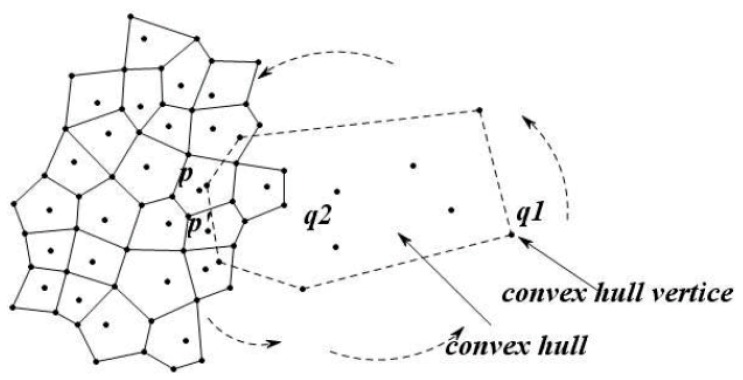
Cut of skyline region based on the convex hull vertices.

Figure 8 shows that the algorithm begins with V(p) including some query node to traverse the dual diagram of the Voronoi diagram which is called the Delaunay graph [30]. It can determine the spatial skyline quickly located in CH(Q) or intersecting with CH(Q) according to Theorem 1 and Theorem 3, and issue the current skyline data to those. The algorithm runs recursively until the initial query node. The process is conducted in a centralized way in the monitor center. Then, the monitor center calculates the rectangle B by the existing SCH, and then cuts the data nodes outside B, lastly, all the accessed data are stored in the data access table Visited, and the center transmits the necessary data to the corresponding sensor nodes in the table SCH for further querying.

**Algorithm 1:** Cutting of the skyline region based on the convex hull vertices**Input:** data node set P, query node set Q**Output:** the skyline set S1: initialize the skyline data table SCH and data access table Visited2: calculates the CH(Q) by the monitor center3: **for** begin with V(p) including some query node q(q2CHv(Q)) and put p into table SCH4: **do** traverse Delaunay graph along each edge of CH(Q)5: **if** V(p’)(p’2P) is adjacent to V(p) and intersecting with CH(Q) or in CH(Q)6: **then** by Theorem 1 and Theorem 3, insert node p into table SCH and table Visited. Issues the current skyline data to those7: **else if** V(p’) is adjacent to V(p) and not intersecting with CH(Q) or not in CH(Q)8: **then** insert node p’ into table Visited9: back to the initial data node p10: **end for**

**Figure 9:**
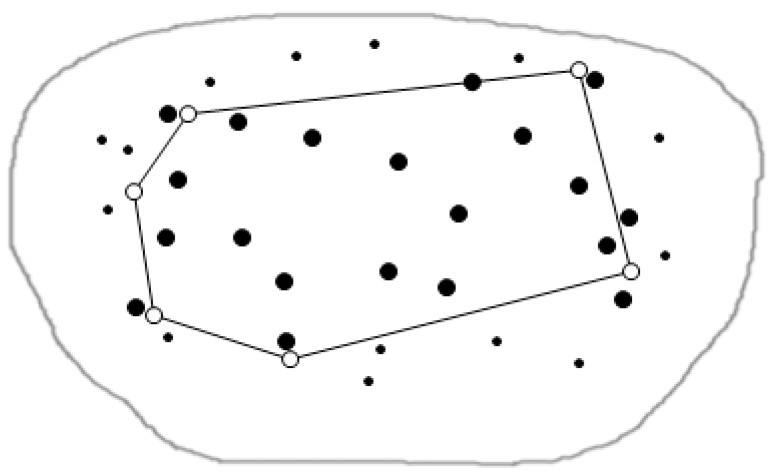
Cutting result for the skyline region.

Figure 9 shows the query results for this phase. The big solid points represent the phase which can query the part of skyline data in the figure, however, small solid points on the outside of the convex hull still have some data not found in the skyline data, so the skyline data query method is put forward in the next section of this article.

- Monotonic function sorting

In order to avoid large comparison times between data and reduce the computing cost, this paper proposes a monotonic function [31] strategy by sorting the data in table SCH. Thus we maintain a list L in which all the skyline data distanced attributes of table SCH are kept. The data in list L are sorted by the sum of the distance between a skyline node and each convex hull vertex in ascending order. When a new node is inserted into L, we only need to conduct the dominated judgement assessment with the node whose distance attribute is less than that of the new one because if the distance attribute of the newly inserted node is less than that of some node in L, it means there is at least a lesser distance than that of the nodes following the new one. Thus, the new node is not dominated by the following nodes and it is not necessary to judge the dominated relation between the other nodes. Via the monotonic function strategy, we can reduce the nodes’ comparison times with lower computing cost and the energy consumption of sensor nodes, meanwhile, we can improve the accuracy and efficiency of the query.

**Figure 10:**
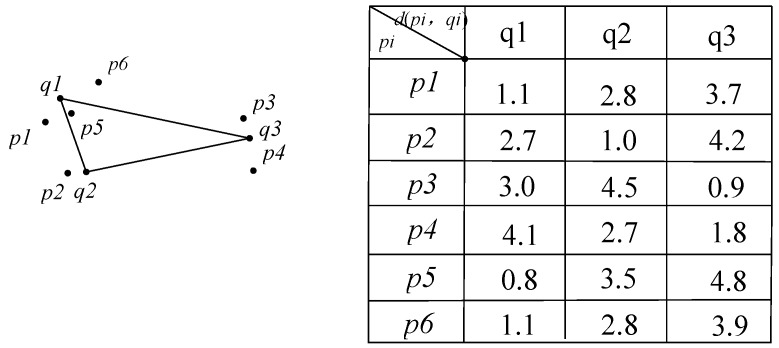
The geography information in sensor node.

As shown in Figure 10, we insert in ascending order into list L all the nodes whose sum of distance to each convex hull vertices is calculated. We can judge if node p1 is located before node p2 via this list. Thus, we can only conduct dominated relationship judgements between node p6 with all the skyline nodes before it in list L. If node p6 is not dominated by the nodes before it, then we insert it into L, then it is the new skyline data. Accordingly, by the monotonic function, we can avoid the dominated relationship judgement between p6 and the nodes following p6. This further reduces the comparison times for data dominance.

## 4. Geometry-Based Distributed Skyline Query

In order to find the sensor nodes which are closer to neighborhoods and have larger air pollution indexes, which are the skyline data we are looking for, research about the remaining spatial skyline queries, parallel queries [32] and distributed queries of general skyline queries of non-spatial attributes in spatial skyline data has been conducted on the basis of the above study. The regional division strategy based on triangulation [33] and sub-region clustering strategy [34] are proposed to provide a basis for spatial skyline queries based on data node trees [35] and parallel queries of non-spatial skyline data.

### 4.1. Regional Division

In order to realize the accuracy and completeness of the query, this paper proposes a regional division strategy based on the triangulation method. In Section 3, after querying partial spatial skyline data via the method of cutting the skyline area based on the convex hull vertices, there are still some spatial skyline nodes outside the convex hull vertices, so we divide the spatial region into several sub-regions, then conduct distributed queries on the remaining spatial skyline data, as well as general queries of these data, so as to realize distributed queries between different sub-regions, and parallel queries in sub-regions. The method can greatly improve the accuracy and efficiency of the queries.

#### 4.1.1. Regional Division Based on Triangulation Method

In order to find the nodes which still exist outside the convex hull, we propose the method of the distributed queries based on the data node tree [22] to find the remaining spatial skyline. Aiming at each closest node, the algorithm builds a data node tree by region and a local queue so that it can traverse all the neighbor nodes which are unvisited and in or intersecting with B of the sub-regions [30]. Finally, the algorithm judges the dominance relationship between nodes by the distributed query to get all the spatial skyline with the distributed query.

**Figure 11:**
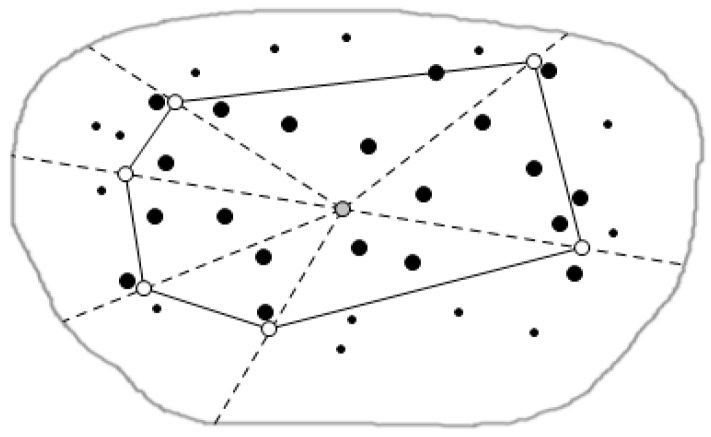
Triangulation diagram.

The monitor center computes the central location R of the CH(Q), and then divides the region into several sub-regions shown in Figure 11 by the triangulation [36] method. We define the data nodes closest to each of the convex hull vertices as the closest nodes. Accordingly, we find each closest node belonging to each triangle area. Here, we define the closest node is the special sensor node which possesses high energy and stronger computing ability.

#### 4.1.2. Clustering in the Sub-Region

**Figure 12:**
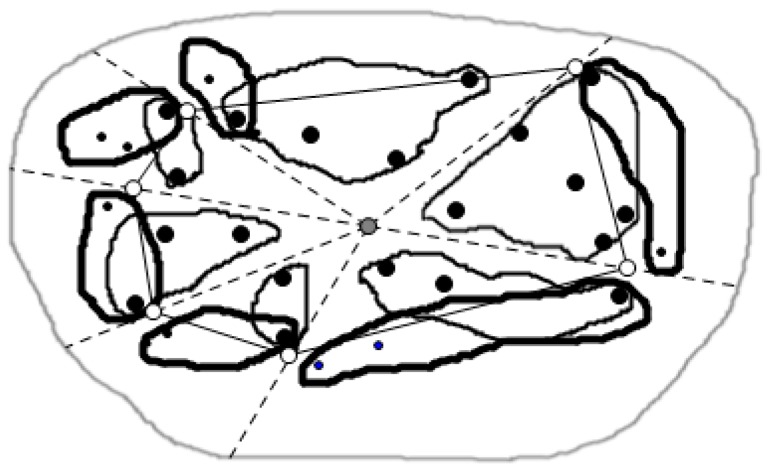
Clustering in the sub-region.

In order to make the skyline query in parallel, this paper has designed a clustering strategy, regarding the closest point as the cluster head node, and the partial spatial skyline obtained from the cutting strategy of the skyline region in each triangle region is classified as one cluster called major cluster, that can return all the spatial skyline ST out of the convex hull by the distributed query method based on the data node tree, and then divide the ST data into an assisted cluster which is shown in Figure 12.

To improve the efficiency of the query, this paper has designed a clustering strategy which divides the triangle region nodes obtained by the triangulation method into a major cluster and an assisted cluster so as to realize the parallel queries. According to the choice of the camera position in the triangulation method, this paper regarded the closest point as the cluster head node, and the partial spatial skyline which was obtained by the cutting strategy of the skyline region in each triangle region is classified as one cluster called major cluster, which can return all the spatial skyline ST out of the convex hull by the distributed query method based on the data node tree, and then divide the ST data into assisted clusters, which is shown in Figure 12. The major cluster and assisted clusters share a common cluster head node in each sub-region. As shown in Figure 12, each little region drawn with a thin line indicates a major cluster and *vice versa*. Data from different sub-regions is transmitted via the respective cluster head nodes.

### 4.2. Distributed Regional Queries

This paper proposes the strategy of distributed regional queries based on the division of the query region. We can query in parallel in each sub-region. Using the spatial skyline query method based on data node trees we can conduct queries in the remaining spatial skyline, meanwhile, it also conducts non-spatial skyline queries on the non-spatial attributes of the spatial skyline, and distributed queries among each sub-region. This paper also proposes a method which can combine the results of each sub-regional skyline query. Firstly, we delete all the dominated results in the sub-region via the cutting method between sub-regions. Then we continue to delete some dominated query results within the sub-region via the cut method. Via the combination of the query results, we can further cut the dominated nodes, improve the accuracy of the queries and reduce the cost of data communication. This paper presents the strategy of distributed regional queries which can quickly return the multi-dimensional attribute skyline results of interest to the user.

#### 4.2.1. Queries in Parallel within Sub-Regions

In Section 3, after cutting the skyline area based on the convex hull vertices, we assign the partial spatial skyline data to the major cluster so as to conduct skyline queries on the non-spatial attributes. However there are some spatial skyline data outsides the convex hull. In order to find these nodes, we propose a spatial skyline query method based on the data node tree to guarantee the completeness and accuracy of the query results, and thus attribute these results to the assisted cluster. The sub-region parallel query method this paper proposes is to conduct skyline queries on non-spatial attributes in the major cluster and to conduct skyline queries on the remaining spatial skyline data. The use of the clustering parallel queries can improve the execution efficiency of the algorithm. Meanwhile this method conducts general skyline queries on the non-attribute data on the basis of the spatial skyline data. It furtherly reduce the time needed for comparing data and reducing the energy consumption of sensor nodes.

(1) Spatial skyline queries based on a data node tree

In order to find the nodes which still exist outside the convex hull, we propose the method of distributed queries based on the data node tree [37] to find the remaining spatial skyline. Aiming at each closest node, the algorithm builds a data node tree by region and a local queue so that it can traverse all the neighbor nodes which are unvisited and in or intersecting with B of the sub-regions [38]. Finally, the algorithm judges the dominance relationship between nodes by distributed queries to get all the spatial skyline data with these distributed queries.

- The generation of the data node tree

**Figure 13:**
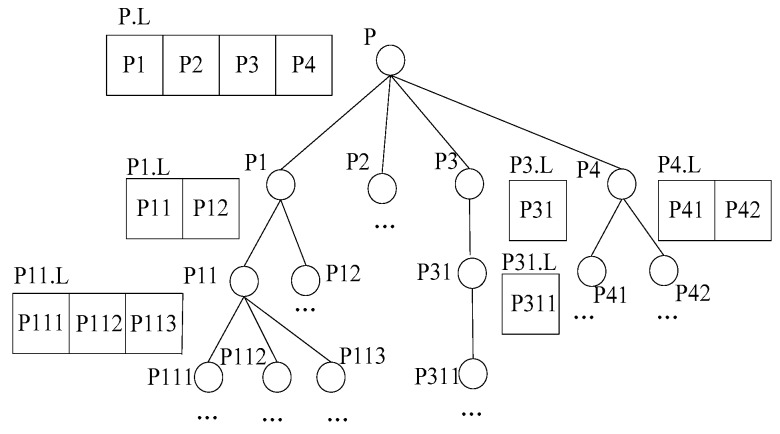
The structure diagram of the data node tree T.

Each closest point receives a skyline data table SCH from the monitoring center and the data node visited table Visited and regards the two tables as the local tables, and maintains a local data node tree T whose root is the closest point and other nodes of the tree correspond to all the data nodes out of the Visited table and in or intersecting with B, as shown in Figure 13.

This paper maintains a local queue L located at each node who possesses neighbors, which is used for the storage of <p, mindist(p, CHv(Q)) > tuple, mindist() is a monotonic function, mindist(p, CHv(Q)) represents the sum of the distances from a data point p to CHv(Q). The specific algorithm is shown as generation data node tree [34] algorithm. The time complexity of the algorithm is O(n).

- skyline queries based on data node trees

At this stage, the algorithm regards the root node of tree T as the parent node, and starts from it to traverse this tree. If the parent node has a local queue L, then the neighbor nodes will be traversed by it, and the dominance relations will be judged with the local skyline data SCH. In this process T will be traversed in depth until the leaf nodes, and then it recursively returns to the next data node of the queue pointer pointing in the upper layer. The process is repeated. The specific algorithm is shown as Algorithm 2. The time complexity of the algorithm is O(n).

**Algorithm 2:** Skyline query based on the data node tree algorithm**Input:** table SCH, table Visited, data node tree T, local queue L, query node set Q**Output**: the spatial skyline data ST based on T1: regard the root node of data node tree T as the father node2: **for** each of the father node **do**3: **if** the father node has the local queue L4: **then** the pointer point to the first record of the father node local queue L, determine dominance relation of the data point p corresponding to the record with the data node in the local table SCH5: **if** P is dominated by the node in table SCH6: **then** regard p as the new father node, then conduct step 27: **if** p is not dominated by the node in table SCH and do not dominate any node in SCH8: **then** insert p to ST, and regard p as new father node, then conduct step 29: **if** p is not dominated by the node in table SCH and dominate the node in table SCH9: **if** p is not dominated by the node in table SCH and dominate the node in table SCH10: **then** insert p to ST, delete the dominated node in SCH, and regard p as new father node, then conduct step 211: **if** the father node do not has the local queue L12: **then** return to the local queue of the father node of p, move the pointer to the next one and regard it as the new father node, then conduct step 213: **end for**

(2) Non-spatial skyline queries

**Figure 14:**
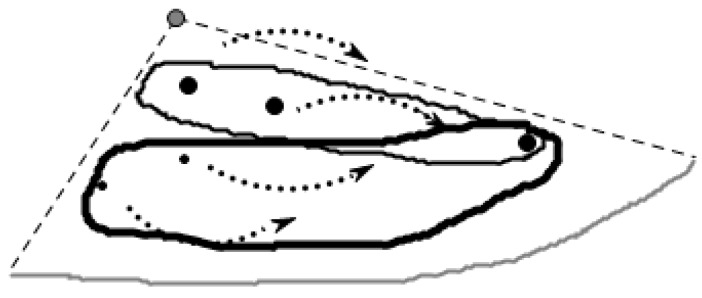
Skyline data collection inside the sub-region.

The algorithm queries the spatial skyline in an assisted cluster, meanwhile, it also conducts the general skyline queries on the non-spatial attributes of the data in the major cluster to get the skyline data Smaj. After the query, the algorithm transmits all the skyline data in the major cluster to the cluster head node. After receiving the remaining spatial skyline found in the assisted cluster, the cluster head node makes the dominance relation judgment between the non-spatial attributes of the spatial skyline in the assisted cluster and the existing skyline data Smaj in the major cluster. Finally, we can find all the skyline data S of the sub-region in the cluster head node of the sub-region. At the same time, each triangle area conducts the above distributed process as shown in Figure 14.

**Figure 15:**
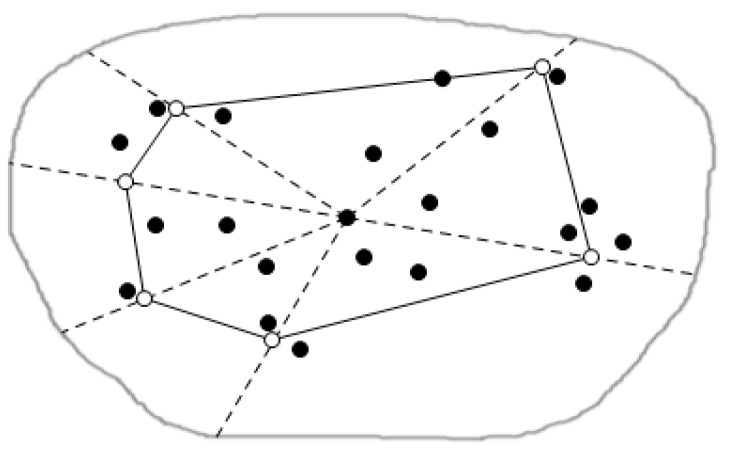
The skyline query results in each area.

The specific algorithm of the above process is shown as Algorithm 3. The time cost of the algorithm is related with the number of nodes in the assisted cluster and the number of general skylines. We suppose P as the number of nodes, S as the number of general skylines, the time complexity of the algorithm is O(|P|(|S|log|CH(Q)| + log|P|)). After the query algorithm, as shown in Figure 15 for the query results, the solid black points in the figure is the skyline query results in each area.

**Algorithm 3:** Syline parallel query algorithm**Input:** table SCH, table Visited, the data node tree T, the local queue L, the query node set Q, the major cluster, the assisted cluster**Output:** the skyline data in sub-region Slocal1: divide the skyline in SCH into the major cluster,2: conduct the distributed query algorithm based on the data node tree algorithm3: divide the data in ST into the assisted cluster and transmit them to the cluster head node4: at the same time, conduct the general skyline query on the non-spatial attribute of the data in the major cluster to get the skyline Smaj5: transmit all the skyline data in the major cluster to the cluster head node6: the cluster head node makes the dominance relation judgment between the non-spatial attributes of the spatial skyline in the assisted cluster with the existing skyline data Smaj in the major cluster7: **then** the cluster head node regards the data that are not dominated by others as the local skyline data of the sub-region Slocal

#### 4.2.2. Query Result Merging in the Inter-Region

This paper proposes a skyline query recovery mechanism which can effectively improve the cut efficiency of the skyline queriesa while reducing the data computation in the skyline queries and reducing traffic in the network of the skyline query results and saving the network energy. This paper proposes the method of cutting among sub-regions and cutting inside sub-regions to conduct domination relation judgments on the skyline data in each region which can quickly cut the non-skyline data, and reduce the volume of data transmission across the network.

(1) Cuts among sub-regions

Each cluster head node establishes a local cluster head node table Sdata when its triangle area finishes calculating all the skyline data, the table includes the tuples of the whole skyline data which is shown in Table 1.

**Figure 16:**
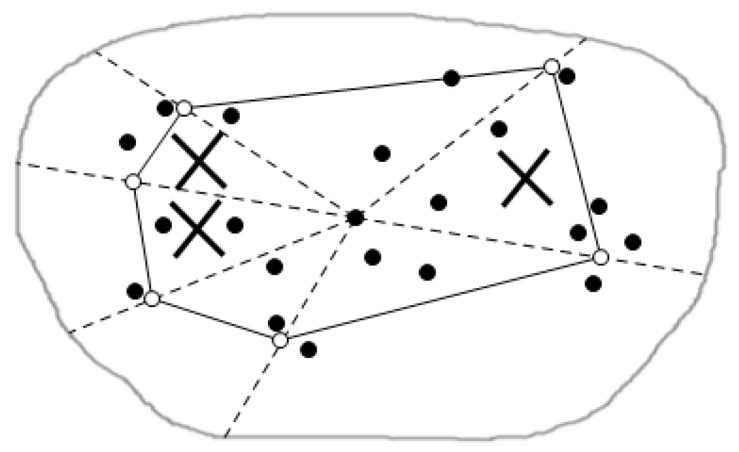
Cuts among sub-regions.

The cluster head node computes maximum and minimum values of each tuple, respectively, and computes the minimum value of the max MIN-max and the minimum value of min MIN-min of the whole tuples in the cluster head node table Sdata, and is supposed to get the interested attribute values which are the smaller the better. If the MIN-max of the cluster head node is smaller than the MIN-min of the other cluster head node, then the sub-regions of the latter are cut totally, as shown in Figure 16, where × represents the sub-regions which are cut.

(2) Cuts inside sub-regions

**Figure 17:**
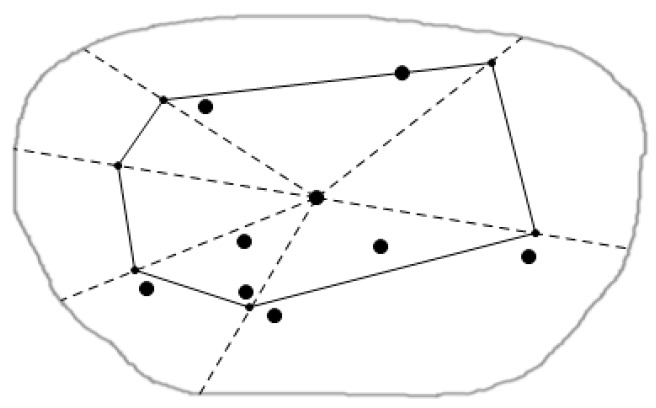
Final results of the skyline query.

In order to improve the skyline query efficiency and reduce the number of data comparisons and the quantity of data transmitted, this paper sets a value of the D-space attribute which is calculated by the multiplication of each attribute in the tuple to represent the dominance ability of a tuple [39]. The calculated value is used to select a tuple possessing the biggest dominance ability as the cut tuple. As the data inside each sub-region does not dominate each other, we only need to judge the dominance relationships between the data in different sub-regions. As shown in Table 2 and Table 3, for two cluster head node Sdata tables, we calculate the D-space value of each tuple value in the table, and if the D-space value of any tuple in one cluster head node table is greater than the D-space value of any tuple in the other cluster head node data table, then we need to further judge the dominance relationship between tuples. For example, If d2’ < d1’ < d2 < d1, then we need to judge the dominance relationship between S2’ with S2 and S1, and still need to judge the dominance relationship between S1’ with S1 and S2 to avoid any non-skyline transmission in the network, and then cut the dominated tuples, and the nodes which are not dominated will be transmitted to the monitor center. As shown in Figure 17, this is the final result of the skyline query. The big solid point represents sensor nodes which are close to residential areas and have higher pollution ability. By this query, we can quickly return the results which meet the demand of users.

### 4.3. The Geometry-Based Distributed Skyline Query Algorithm

The algorithm conducts the general skyline queries on the non-spatial attributes of the data in the major cluster to get the skyline Smaj, transmits all the skyline data in the major cluster to the cluster head nodes, the cluster head nodes perform the dominance relation judgment between the non-spatial attributes of the spatial skyline in assisted clusters with existing skyline data Smaj in the major cluster, and the cluster head nodes regard the data that are not dominated by others as the local skyline data of the sub-region. The collection process can cut all the local skyline of the dominated sub-region with the cuts among sub-regions, then cut all dominated local skyline by the cuts inside sub-regions to get the final skyline data and transmit them to the monitor center. The specific algorithm is shown as Algorithm 4.

**Algorithm 4:** Geometry-based distributed spatial skyline query algorithm**Input:** data node set P, query node set Q**Output:** the skyline set S1: divide the spatial region by Voronoi diagram2: conduct cut of skyline region based on convex hull vertices, acquire part of the spatial skyline data SCH, and transmit them to each nodes, and divide them into the major cluster3: divide the space region into several triangle sub-region by the triangulation4: **for** each closest node, conduct generation data node tree algorithm, conduct the general skyline query on the data in major cluster, and return the result to the cluster head node5: conduct distributed query algorithm.6: conduct skyline parallel query algorithm7: conduct the process of cut among sub-region, use the threshold to cut the dominated sub-region8: conduct the process of cut inside sub-region, transmit the nodes which are not dominated to the monitor center

The time cost of the division of space region using the Voronoi diagram is O(nlogn + 2n − 5), then the cost of the triangulation on the division area is O(nlogn), and the cost of the execution of the skyline query is associated with the number of the data node P in the triangle area, and the time cost is O(|P|(|S|log|CH(Q)| + log|P|)), so the whole time complexity is O(nlogn + n).

## 5. Experimental Performance Analysis

This paper proposes the method of geometry-based distributed spatial skyline query, it solves the issue whereby existing methods cannot adapt to provide efficient specific queries on the spatial distance of any region. The algorithm can reduce communication overhead in the network and improve the network life of monitoring systems by using geometry-based distributed spatial skyline queries. This paper discusses six aspects concerning the number of dominance comparisons between nodes, the number of query nodes with dominance comparisons, the sensor energy consumption, the response time of the query nodes, the execution efficiency percentage and the collection efficiency of the skyline. This paper compares them with the methods used in wireless sensor networks. At present, spatial and non-spatial skyline methods can be applied to query specific environmental monitoring regions. Non-spatial skyline queries also shows high response time and node recovery time efficiency. Therefore, we designed some corresponding comparative experiments for them. Voronoi-based Spatial Skyline (VS2) [19] and Enhanced Spatial Skyline (ES) [20] are the spatial skyline query methods, the Top-down filtering method of FIST (TF) [40] and Energy-Efficient Evaluation of Multiple Skyline Queries over a Wireless Sensor Network (EMSE) [41] are the non-spatial skyline query methods. We simulate a data set consisting of 1000 uniform distributed random locations in three-dimensional space. The experiment uses synthetic data as a standard test data set [32,33,34,42,43,44,45,46]. The value in spatial attributes of the nodes is in the range of [0, 1]. The hardware parameters used in the experiment are shown in Table 4.

The parameters used in the experiment are shown in Table 5.

### 5.1. Number of Data Nodes with Dominance Comparisons

**Figure 18:**
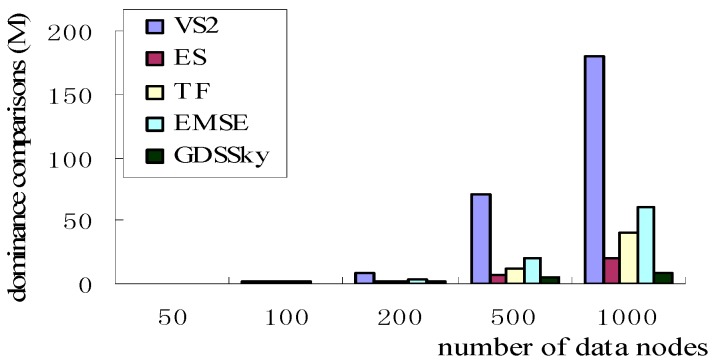
Number of data nodes and dominance comparisons.

As shown in Figure 18, all the VS2 skylines will undoubtedly increase the dominance comparisons. The monotone function used in GDSSky sorts the space distance, which only needs to compare the dominance relation with the partial skyline, while the second stage of ES starts from a point to recursively traverse the neighbor nodes and judges dominance relations with all existing skyline nodes. The TF strategy sets local or global filters in the sensor node, and the filter computation brings a lot of overhead, though the data detected by the sensor node is not beyond the range of super-cube, however, it cannot judge whether the node is the skyline or not because of the change of the detected data of the other nodes, however, it needs to acquire the detected data of this node, which will result in the more domination relationship comparisons. The EMSE algorithm uses the global and local two processes, allocates a signature for each tuple in the process of local optimization to indicate the query where the tuple belongs to, all tuples can only participate in a query, when the data in the network changes, dominance relation the tuple is likely to be falsely judged. However, the paper uses the point location queries mechanism without dominance comparisons between nodes to get most local skyline data. The rectangular B can reduce the dominance comparisons between nodes at the later stage. The more the number of sensor nodes is, the more obvious the effect.

### 5.2. Number of Query Nodes with Dominance Comparisons

**Figure 19:**
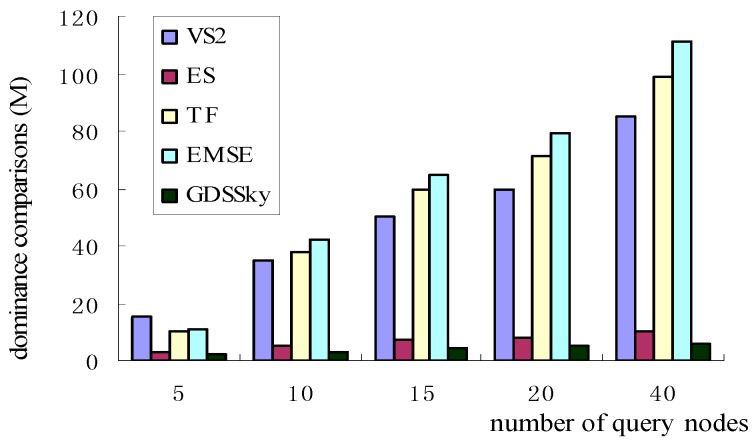
Number of query nodes with dominance comparisons.

Figure 19 shows the relationship between the number of query nodes with the dominance comparisons. Figure 18 and Figure 19 are similar, with the increase of the number of query nodes, the number of nodes dominating comparisons increases relatively steadily, because all the above three spatial-based algorithms—VS2, ES, GDSSky—use CH(Q) instead of Q, and the size of CH(Q) grows slower than the size of Q. In the two algorithms based on the non-spatial—TF, EMSE—all sensor nodes are required to participate in the query, that is, each dimension of all sensors including multi-dimensional spatial attributes must be involved in the judgment of domination relations, which will no doubt greatly increase the dominance relation comparisons. The VS2 algorithm starts from a data node to judge the relationship with other nodes. This also increases the comparisons between the nodes. The GDSSky algorithm we have proposed in this paper, firstly, finds all the interesting spatial skyline by the spatial skyline query method, and cuts off amounts of non-skyline data, then it uses the non-spatial attributes of the spatial skyline to conduct the general skyline queries among the sub-regions and inside the sub-regions to cut respectively, and also reduces the number of dominance comparisons.

### 5.3. Sensor Energy Consumption

**Figure 20:**
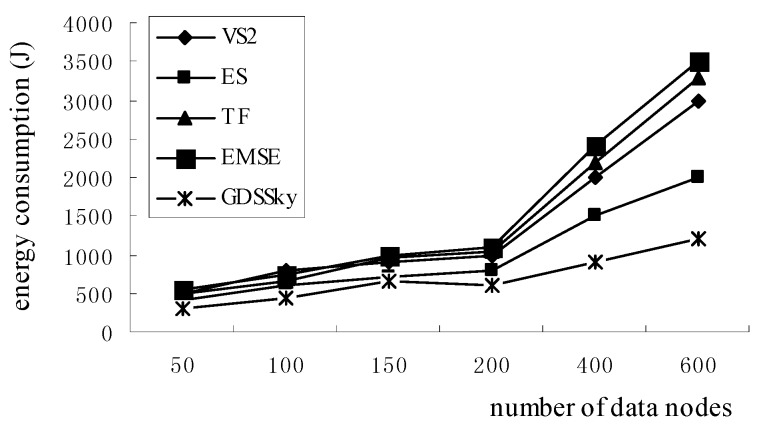
Sensor energy consumption.

As shown in Figure 20, the energy consumption in GDSSky is less than that of the other four methods, because the VS2 algorithm transmits information to one node and calculates the dominance relation in the overall recursion query phase and the ES algorithm in the second phase transmits to the same node information to determine the dominance relation, and both consume energy. All the sensor nodes and all the attributes of the algorithms of TF and EMSE are required to participate in the filter and the dominance relation judgment, and the multi-dimensional spatial attributes produces an energy consumption which increases as the number of data nodes increases. Our strategy proposes a method to acquire the skyline on the spatial attributes so that it can cut a large part of the non-skyline, which can reduce the energy cost in the network. This paper cuts part of the non-spatial skyline first, then based on it we conduct a general skyline query to reduce the dominance comparisons between nodes and the data transmission, which reduces the energy consumption.

### 5.4. Effect of the Number of Query Nodes on the Response Time

**Figure 21:**
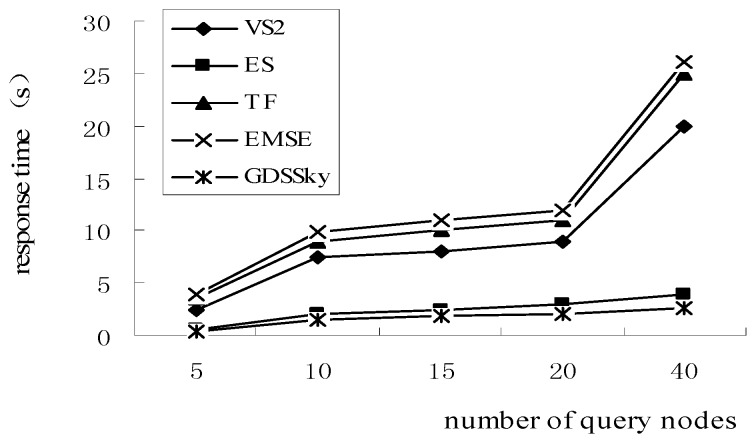
Relation between number of query nodes and response time.

Figure 21 shows the effect of the number of query nodes on the response time. The GDSSky outperform the VS2 algorithm, TF algorithm and EMSE algorithm. Because they all reduce the dominance relation judgment between nodes which are in or intersecting with CH(Q), they can reduce lots of the dominance comparisons between nodes. As our algorithm is executed in a distributed manner, compared with the ES algorithm is has a shorter response time. Our method uses the skyline parallel query strategy in the sub-region to query the skyline in the major cluster. Meanwhile, it queries the remaining spatial skyline data, which can improve the response time. In the TF algorithm, the base station can determine the skyline result after obtaining relevant detected data of the sensor nodes, so its query response time is long. In the EMSE strategy, with the increase of query nodes, as the distances of each sensor node to each query nodes must be calculated, this greatly increases the response time.

### 5.5. Execution Efficiency Percentage

**Figure 22:**
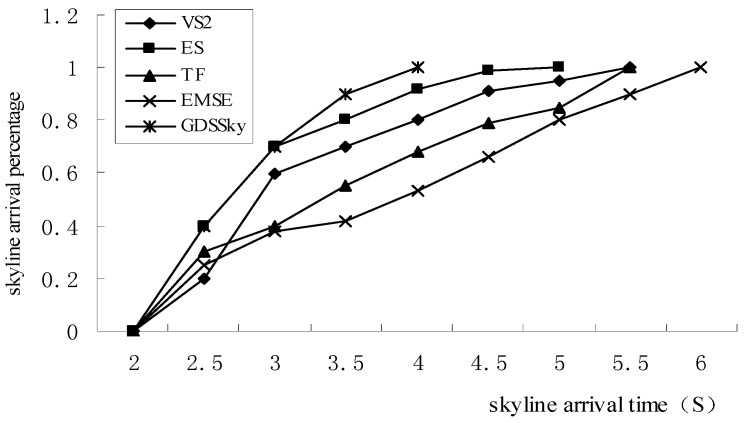
Execution efficiency percentage.

Figure 22 shows that our method proposes the GDSSky method that acquires the remaining skyline at sub-stations after receiving the partial skyline, so it can obtain all spatial skylines earlier than the ES algorithm. The VS2 algorithm executes from one node recursively, so it gets a linear internal skyline, and the arrival time of the external skyline is relatively slow. Meanwhile the TF and EMSE algorithms calculate the skyline of all interested attributes together, due to the fact the dominance relationship comparisons are too many, so the data needs to be continuously transmitted within the network, so the skyline data is obtained relatively slowly.

### 5.6. Skyline Collection Efficiency

**Figure 23:**
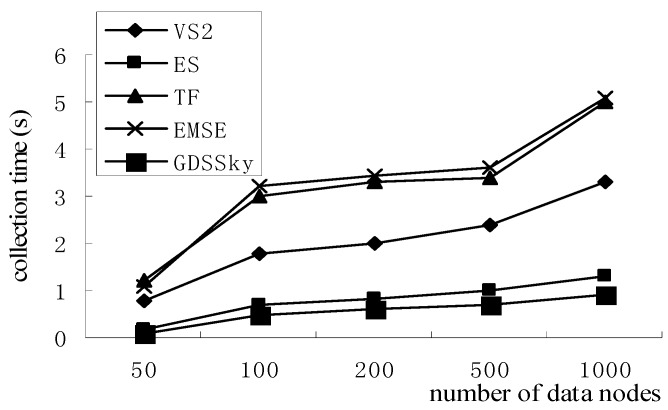
Collection efficiency of the skyline.

Figure 23 shows the effect of the number of sensor nodes on the skyline collection time, which grows with the increase of the number of sensor nodes. However, the skyline collection time of our method is the least, because our method will cut more non-spatial skyline with the increasing data nodes by the spatial skyline query method. Based on it, the skyline query time will be short. Our method conducts the queries and collection at the same time, querying the skyline on the non-spatial attributes of the spatial skyline at the same time. Our method uses a clustering strategy to return all the data back to the cluster head node so that it improves the efficiency. At the collection stage, it uses the cuts among the sub-regions to cut the amount of non-skyline quickly, and then utilizes cuts inside the sub-region to accurately cut the dominated nodes by judging the dominance relation through the value of the D-space threshold, and at last transmits them to the monitor center, while the VS2 algorithm judges the dominance relationships one by one, then it uploads data to the monitor center so the collection efficiency is low. Both ES and VS2 strategies completely query and collect the spatial attributes, and then conduct the general skyline queries. These strategies have low efficiency. While all the attributes of the TF and EMSE algorithms take part in the dominance relation judgment, the growing number of comparisons increases the recovery time.

## 6. Conclusions

To address environmental problems, this paper proposes a geometry-based distributed skyline query strategy based on the characteristics of spatial data and aiming to solve the deficiencies of the existing skyline query strategies. We design a clustering strategy for the parallel execution of general skyline queries on the non-spatial attributes of the spatial skyline, and conduct the spatial skyline queries on the remaining spatial skyline data which are still not found by the method of the tree at the same time, so it will be conducted in parallel. This paper proposes a distributed execution between different sub-regions. Finally we propose the use of cuts among sub-regions and cuts in sub-regions for the collection.

To solve the problem that the existing spatial and non-spatial skyline query strategies have large energy consumption and poor real-time performance in querying the sensor network, this paper used the concept of the geometry-based spatial skyline, which is based on the query problem of environmental pollution monitoring systems, to reduce the network communication consumption, and increase the network lifetime of the monitoring system.

## Figures and Tables

**Table 1:** Format of the data tuples.

Node ID	Attribute 1 Value	Attribute 2 Value	Maximum Value	Minimum Value	Multiplication of Each Attribute
ID	Attribute 1	Attribute 2	max	min	D-space

**Table 2:** Data table S1 of cluster nodes.

ID	Attribute 1	Attribute 2	Max	Min	D-Space
S1	v1	v11	m1	m11	d1
S2	v2	v22	m2	m22	d2

**Table 3:** Data table S1’ of cluster nodes.

ID	Attribute 1	Attribute 2	Max	Min	D-Space
S1’	v1’	v11’	m1’	m11’	d1’
S2’	v2’	v22’	m2’	m22’	d2’

**Table 4:** Hardware parameters.

Hardware	Parameter
CPU	Pentium 4 (3.2 GHz)
Memory	4 GB
Disk	1 TB

**Table 5:** Experimental Parameters.

Parameter	Setting
Dimensionality	3
Dataset cardinality	50, 100, 200, 500, 1000
Distribution of data points	Independent
The number of points in a query	5, 10, 15, 20, 40
